# Humanizing the intensive care unit

**DOI:** 10.1186/s13054-019-2327-7

**Published:** 2019-01-28

**Authors:** Michael E. Wilson, Sarah Beesley, Amanda Grow, Eileen Rubin, Ramona O. Hopkins, Negin Hajizadeh, Samuel M. Brown

**Affiliations:** 1 Division of Pulmonary and Critical Care Medicine, Mayo Clinic, Rochester, MN USA; 2 Robert D. and Patricia E. Kern Center for the Science of Health Care Delivery, Mayo Clinic, Rochester, MN USA; 3 Biomedical Ethics Program, Mayo Clinic, Rochester, MN USA; 4 Center for Humanizing Critical Care at Intermountain Healthcare, Murray, UT USA; 5 Pulmonary and Critical Care Medicine, Intermountain Medical Center, Murray, UT USA; 6 Pulmonary and Critical Care Medicine, University of Utah School of Medicine, Salt Lake City, UT USA; 7 ICU Patient and Family Advisory Council, Intermountain Medical Center, Salt Lake City, USA; 8 ARDS Foundation, Northbrook, IL USA; 9 Department of Psychology and Neuroscience, Brigham Young University, Provo, UT USA; 10 Division of Pulmonary Critical Care Medicine, Zucker School of Medicine at Hofstra/Northwell, Manhasset, NY, USA; 11 Division of Medical Ethics and Humanities, University of Utah School of Medicine, Salt Lake City, UT USA; 12 Shock Trauma ICU, Intermountain Medical Center, Murray, UT USA

## Introduction

In the midst of trying to correct organ failures, clinicians may neglect to carefully consider what the patient is experiencing: to be on the brink of death, be unable to speak, be stripped naked, have strangers enter the room and simultaneously do things to their bodies without explanation, have tubes inserted into multiple orifices, have their arms restrained, hear a cacophony of disorienting bedside alarms whose meaning lies beyond them, and to be poked, and prodded—all while family is torn away. Compounding these facts, patients often have no memory or understanding of how they ended up in this horrifying situation. Encephalopathy makes it difficult for patients to make sense of the myriad painful stimuli they encounter. Patients and families must surrender all control.

**Fig. 1:**
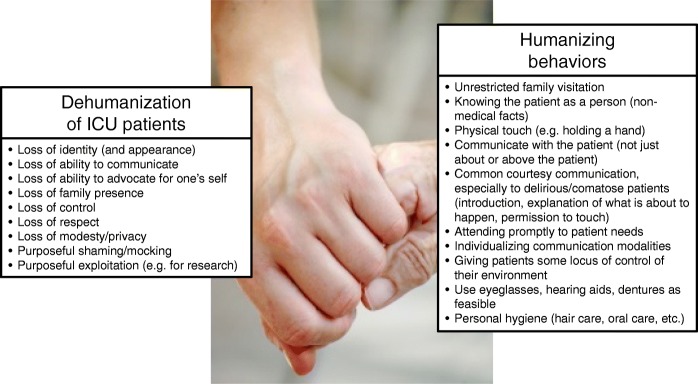
Dehumanization and humanization of intensive care unit patients

In all of this perceived chaos, some patients who experience critical illness may experience a loss of their humanity in the process. This loss of humanity may come in many forms, including the loss of personal identity, control, respect, privacy, and support systems, and is referred to as dehumanization (Fig. 1). Dehumanization consists of treating someone as an “object” rather than a “person” and is often associated with failures to honor dignity [1].

## What does dehumanization of ICU patients look like?

ICU patients experience a devastating loss of personal identity. Instead of being identified by their names, personalities, interests, families, and cultures, patients are reduced to their room numbers, their diseases, or the treatments they receive—e.g., “512, resolving sepsis.” Personal identities are also lost by uniform standardized clothing (the hospital “gown”), inability to communicate, delirium, impaired hygiene, and absence of eyeglasses and hearing aids.

Patients also lose their ability to control their environment, govern their own actions, and advocate for themselves—often made worse by loss of consciousness. When faced with a patient who is unconscious or cannot talk, physicians may enter the patient’s room without introduction, proceed to move parts of the patient’s gown and touch the patient without notice, speak to the nurse *about* what is going on with the patient, and leave the patient’s room all without uttering a single word *to* the patient. Patients with altered consciousness often report traumatic memories of their ICU stays and feeling like their bodies were not even their own anymore.

Furthermore, patients often lose their family as they are escorted to the “waiting” room. In essence, restrictive visitation systematically removes from the bedside the world experts on that particular patient, in addition to pulling away the most central support system of most patients—all at the most vulnerable point in their lives.

## Why does dehumanization of ICU patients occur?

High workload and burnout may lead healthcare team members to become desensitized to the human aspects of critical illness [2]. Policies and cultures of many ICUs (such as restrictive visitation) promotes dehumanization by further taking control from patients and families [3]. Fragmented care delivery models (shift work) may also unintentionally prevent ICU physicians from getting to know their patients as people. Clinicians may not remember that patients who seem unconscious may feel and remember what they are experiencing. While ICU clinicians may have expert knowledge of critical care, few have experienced life as an ICU patient or thought carefully about what that experience might feel like. For those clinicians who have experienced being a patient, the insider experience has taught them the importance of family bedside presence, physical touch such as holding a hand, and calm words of explanation, safety, and support [4].

## How can we consider the humanity of the person in the bed?

Several considerations may improve the humane and respectful treatment of ICU patients. First, we recommend patient-centered family visitation—the only routine restrictions should be driven by patient request. Open visitation has been associated with less anxiety, less PTSD, less agitation, shorter length of ICU stay, higher patient/family satisfaction, and even improved patient safety [5–7]. Second, we recommend speaking to all ICU patients—even those who are delirious, comatose, or unable to speak. When entering the patient’s ICU room, healthcare team members should introduce themselves, their role, and what is happening. For example, a physician could shake the patient’s hand and say, “Hello Mr. Jones [ideally the patient’s preferred name], this is Doctor Stuart, the second in command [i.e., ICU fellow] on your ICU team. I am here to check your heart and lungs this morning. I would like to remove the gown off of your chest and listen to your heart with my stethoscope.” Strategies to reorient patients and explain what is happening have been associated with less delirium, shorter length of mechanical ventilation, and less sedative use [8, 9]. Third, we recommend minimizing the effects of altered consciousness and impaired mobility, including individualized efforts to minimize sedation, reduce delirium, and promote early physical therapy/mobility [10]. Fourth, we recommend learning something about the patient as a person. Things such as a “get to know me board” or photographs of pre-ICU life may help clinicians better understand the patient as a person. Some of us begin family meetings with the open-ended request, “Tell me about him as a person. What do you think are some great stories about her that will help us understand her better as a person?”

## Implications of dehumanization

Key aspects of patients’ illnesses as well as the behaviors/attitudes of healthcare teams contribute to the dehumanization of ICU patients. Not treating patients as humans risks serious consequences for patients’ physical and mental wellbeing, both during critical illness as well as their recovery long after surviving. When clinicians fail to consider the personal identities of their patients, there risks potential biases in how clinicians prognosticate and ultimately engage in decision making about life support withdrawal. Efforts to humanize the ICU may have benefits in boosting patients’ attitudes and engagement in their own well-being. Understanding and addressing patient-, clinician-, and system-level factors that contribute to dehumanization of ICU patients represent significant areas necessary for investigation and intervention in our efforts to advance the delivery of high-quality critical care.
